# Impacts of Oxidative Stress and PI3K/AKT/mTOR on Metabolism and the Future Direction of Investigating Fucoidan-Modulated Metabolism

**DOI:** 10.3390/antiox11050911

**Published:** 2022-05-06

**Authors:** Jun-Ping Shiau, Ya-Ting Chuang, Yuan-Bin Cheng, Jen-Yang Tang, Ming-Feng Hou, Ching-Yu Yen, Hsueh-Wei Chang

**Affiliations:** 1 Department of Surgery, Kaohsiung Municipal Siaogang Hospital, Kaohsiung 81267, Taiwan; drshiaoclinic@gmail.com; 2 Division of Breast Oncology and Surgery, Department of Surgery, Kaohsiung Medical University Hospital, Kaohsiung Medical University, Kaohsiung 80708, Taiwan; mifeho@kmu.edu.tw; 3 Department of Biomedical Science and Environmental Biology, College of Life Science, Kaohsiung Medical University, Kaohsiung 80708, Taiwan; u107023007@gap.kmu.edu.tw; 4 Department of Marine Biotechnology and Resources, National Sun Yat-sen University, Kaohsiung 80424, Taiwan; jmb@mail.nsysu.edu.tw; 5 School of Post-Baccalaureate Medicine, Kaohsiung Medical University, Kaohsiung 80708, Taiwan; reyata@kmu.edu.tw; 6 Department of Radiation Oncology, Kaohsiung Medical University Hospital, Kaoshiung Medical University, Kaohsiung 80708, Taiwan; 7 Department of Oral, Maxillofacial Surgery Chi-Mei Medical Center, Tainan 71004, Taiwan; 8 School of Dentistry, Taipei Medical University, Taipei 11031, Taiwan; 9 Center for Cancer Research, Kaohsiung Medical University, Kaohsiung 80708, Taiwan

**Keywords:** oxidative stress, PI3K, AKT, mTOR, metabolism, anticancer, fucoidan

## Abstract

The critical factors for regulating cancer metabolism are oxidative stress and phosphoinositide-3-kinase/AKT serine-threonine kinase/mechanistic target of the rapamycin kinase (PI3K/AKT/mTOR). However, the metabolic impacts of oxidative stress and PI3K/AKT/mTOR on individual mechanisms such as glycolysis (Warburg effect), pentose phosphate pathway (PPP), fatty acid synthesis, tricarboxylic acid cycle (TCA) cycle, glutaminolysis, and oxidative phosphorylation (OXPHOS) are complicated. Therefore, this review summarizes the individual and interacting functions of oxidative stress and PI3K/AKT/mTOR on metabolism. Moreover, natural products providing oxidative stress and PI3K/AKT/mTOR modulating effects have anticancer potential. Using the example of brown algae-derived fucoidan, the roles of oxidative stress and PI3K/AKT/mTOR were summarized, although their potential functions within diverse metabolisms were rarely investigated. We propose a potential application that fucoidan may regulate oxidative stress and PI3K/AKT/mTOR signaling to modulate their associated metabolic regulations. This review sheds light on understanding the impacts of oxidative stress and PI3K/AKT/mTOR on metabolism and the future direction of metabolism-based cancer therapy of fucoidan.

## 1. Introduction

Diverse metabolisms are essential for cancer cell proliferation by regulating redox homeostasis, energy, and biosynthesis [1,2,3,4]. Several factors that modulate metabolism may improve the anticancer therapeutic effects. Oxidative stress and phosphoinositide-3-kinase/AKT serine-threonine kinase/mechanistic target of the rapamycin kinase (PI3K/AKT/mTOR) are important in regulating several kinds of metabolisms in cancer cells [5,6,7,8,9,10].

In this review, we focus on glycolysis (Warburg effect), pentose phosphate pathway (PPP), fatty acid metabolism, tricarboxylic acid cycle (TCA) cycle, glutaminolysis, and oxidative phosphorylation (OXPHOS). The relationship and interaction between oxidative stress, PI3K/AKT/mTOR, and associated metabolisms are summarized.

Several natural products show differential responses to cancer and normal cells and cause selective killing effects on cancer cells [11,12,13,14]. These drug-induced selective killing effects are associated with elevated oxidative stress generation in cancer cells compared to normal cells. Moreover, the PI3K-AKT-mTOR pathway exhibits a diverse function for regulating proliferation, metabolism, and metastasis [15,16,17,18,19]. Several cancer cells show higher expressions of AKT than normal cells [20,21,22,23,24], suggesting that PI3K-AKT-mTOR may have differential responses between cancer and normal cells. Accordingly, natural products with oxidative stress or PI3K-AKT-mTOR modulating ability are expected to provide potent anticancer candidates.

Fucoidan, a brown alga-derived polysaccharide, is a safe food supplement with suitable nutraceutical characteristics [25]. Recently, several chemopreventive and antiproliferation effects of fucoidan were reported [25], but their mechanisms and connections to metabolism have not been fully investigated yet. The functions of oxidative stress and PI3K/AKT/mTOR in fucoidan are summarized here. Although their impacts on metabolism remain unclear, the potential application of fucoidan-modulated metabolism is discussed.

This review aims to illustrate existing knowledge of individual (Section 2 and Section 3) and interacting (Section 4) effects of oxidative stress and PI3K-AKT-mTOR as well as their impact on fucoidan treatments (Section 5). We also hypothesize that these metabolic regulations may act on fucoidan treatment (Section 6). Finally, we provide a novel rationale that oxidative stress and PI3K/AKT/mTOR signaling may play a vital role in metabolism-related cancer therapy using fucoidan.

## 2. Oxidative Stress and Its Associated Metabolisms

**Figure 1:**
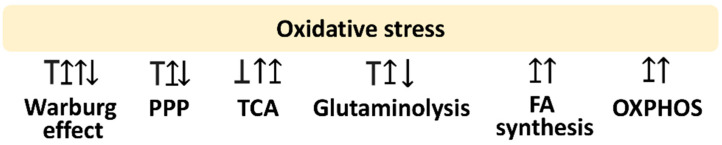
Oxidative stress and its associated metabolisms. Arrow indicates activation; T indicates inhibition; T with arrow indicates inhibition leading to activation. Abbreviations: PPP, pentose phosphate pathway; TCA, tricarboxylic acid cycle; FA, fatty acid; OXPHOS, oxidative phosphorylation. These effects are summarized in the reports mentioned in Section 2.1, Section 2.2, Section 2.3, Section 2.4, Section 2.5, Section 2.6. Different studies reported differential regulations to these metabolisms by modulating oxidative stress. Various reports show different responses to oxidative stress for the same metabolism.

Oxidative stress is a modulator for metabolism. This review is mainly concerned with glycolysis, PPP, fatty acid synthesis, TCA cycle, glutaminolysis, and OXPHOS. The following sections provide the impact of oxidative stress on regulating these metabolisms, which is summarized in Figure 1.

### 2.1. Relationship between the Warburg Effect and Oxidative Stress

Cancer cells prefer aerobic glycolysis for lactate production (namely Warburg effect) over oxidative phosphorylation because lactate generates several metabolites for supporting cancer cell proliferation [26]. Moreover, cancer cells take up more glucose by upregulating glucose transporting receptors such as glucose transporter (GLUT) [27].

The Warburg effect shows the crosstalk relationship with oxidative stress. Oxidative stress may activate the Warburg effect, while the Warburg effect may activate or inactivate oxidative stress. Several reports concerning these relationships were described as follows (Figure 1).

#### 2.1.1. Warburg Effect May Inhibit Oxidative Stress

The Warburg effect may benefit cancer cell proliferation by reducing cytotoxic oxidative stress generated from aerobic respiration [5]. This oxidative stress-reducing ability is proposed to inhibit the energy dependence on mitochondrial OXPHOS, the main pool of oxidative stress. The end product of glycolysis is pyruvate; it converts to acetyl-CoA to enter the TCA cycle and processes OXPHOS. In comparison, pyruvate is converted to lactate by the Warburg effect and bypasses OXPHOS, decreasing oxidative stress [6].

In the case of Warburg effect inhibition, OXPHOS is activated. By the example of glycolysis inhibition by targeting glucose-6-phosphate isomerase (GPI; glucose-6-phosphate → fructose-6-phosphate), the energy flow is mainly attributed to OXPHOS [28]. Dichloroacetate (DCA), a mitochondrial pyruvate dehydrogenase kinase 1 (PDK1) inhibitor, also shows similar results in inhibiting the Warburg effect and switching to OXPHOS [29]. Subsequently, OXPHOS produces more oxidative stress and induces apoptosis in cancer cells [30]. Accordingly, the Warburg effect can inhibit oxidative stress-associated responses such as apoptosis of cancer cells. These results emphasize the rationale that suppressing the Warburg effect and switching to OXPHOS offers oxidative stress to inhibit the proliferation of cancer cells [31] (Figure 1).

#### 2.1.2. The Warburg Effect May Induce Oxidative Stress

In normal cells, glycolysis generates pyruvate to enter the TCA cycle, producing several antioxidant metabolites such as citrate, malate, and oxaloacetate. Moreover, fumarate, one of the TCA metabolites, can enhance NFE2-related factor 2 (NRF2) antioxidant signaling [32]. During this processing, NADH is generated, and it is converted to nicotinamide adenine dinucleotide phosphate (NADPH) by nicotinamide nucleotide transhydrogenase (NNT). Subsequently, NADPH is converted to the antioxidant glutathione (GSH) by the catalyzation of glutathione reductase (GSR) [32]. Accordingly, these signaling pathways contribute to the antioxidant potential of normal cells.

In contrast, cancer cells skip the TCA and OXPHOS pathways but favor the Warburg effect to downregulate antioxidant signaling [32]. As a result, cancer cells may show high oxidative stress. Therefore, the Warburg effect induces oxidative stress in cancer cells (Figure 1).

#### 2.1.3. Oxidative Stress May Induce the Warburg Effect

Cancer cells may show adaptation to high oxidative stress. In response to oxidative stress, the Warburg effect of cancer cells is activated [6]. The oxidative stress may be induced by downregulating antioxidant signaling such as AMP-activated protein kinase (AMPK)-responsive antioxidant response [33]. Mitochondrial reactive oxygen species (ROS) may influence the Warburg effect of AMPK-defective cancer cells [34]. Therefore, the ROS-modulating ability on the Warburg effect shows a bidirectional regulation (Figure 1).

### 2.2. Relationship between the PPP and Oxidative Stress

In addition to activating the Warburg effect, high oxidative stress of cancer cells may exhibit an alternative choice. When oxidative stress affects cancer cells, the Warburg effect is initially induced, and then it switches to the pentose phosphate pathway (PPP) if the oxidative stress is prolonged. Consequently, the NADPH generated from PPP scavenges ROS and reduces oxidative stress [6]. PPP generates NADPH to mitigate oxidative stress mainly derived from oxidative phosphorylation [35], but cancer cells still maintain high non-toxic oxidative stress for their malignant proliferation [6,36].

In the example of thyroid cancer cells, metabolomic results show high expressions of the PPP signaling pathway [37]. Inhibitors for the key enzymes of PPP (glucose-6-phosphate dehydrogenase (G6PD) and transketolase), such as 6-aminonicotinamide and oxythiamine, exhibit antiproliferation, accompanied by inducing ROS, apoptosis, and endoplasmatic reticulum stress [37]. Accordingly, PPP exhibits an oxidative stress-suppressing function in cancer cells. Targeting PPP causes oxidative stress to kill cancer cells.

In contrast, PPP may induce oxidative stress generation of drug treatment. The example of itaconic acid shows that PPP induction enhances oxidative stress and suppresses inflammation and bacterial growth [38]. Therefore, the ROS-modulating effects on PPP demonstrate a complex regulation (Figure 1).

### 2.3. Relationship between the TCA Cycle and Oxidative Stress

The TCA cycle exhibits oxidative stress-modulating functions. TCA cycle enzymes are sensitive to ROS [39]. ROS mainly targets aconitase and α-ketoglutarate dehydrogenase (oxoglutarate dehydrogenase; OGDH) to inhibit the TCA cycle [40]. ROS can suppress aconitase to pause α-ketoglutarate generation [41]. ROS can also inactivate OGDH to shut down the TCA cycle [40]. In contrast, malate dehydrogenase (MDH) inhibition induces ROS generation in breast cancer cells kept under hypoxia [42].

Moreover, oxidative stress regulation on the TCA cycle was demonstrated in breast cancer cells and tumor tissues exhibiting low aconitase 2 (ACO2) [43]. ACO2-overexpression causes antiproliferation to breast cancer cells [43], accompanied by a decreasing lactate level, increasing acetyl-CoA level, activating citrate synthase (CS), rising levels of TCA cycle metabolites for citrate, α-ketoglutarate, fumarate, and inducing mitochondrial superoxide [43]. Therefore, the ROS-modulating effects on the TCA cycle are complex and show a complex regulation (Figure 1).

### 2.4. Relationship between Glutaminolysis and Oxidative Stress

Glutaminolysis is a supporting step for the anabolic pathway to replenish the TCA metabolite α-ketoglutarate by converting glutamine to glutamate and becoming α-ketoglutarate, which is catalyzed by glutaminase (GLS) and glutamate dehydrogenase (GLUD1, GDH1) [44,45].

The function of glutaminolysis is to maintain redox homeostasis [46] by participating in antioxidant production to reduce oxidative stress [44,47,48]. Glutaminolysis provides several reducing powers such as reduced glutathione (GSH), NADPH, and α-ketoglutarate and decreases oxidative stress [46]. Moreover, glutaminolysis-derived α-ketoglutarate can run the TCA cycle and become fumarate, which controls oxidative stress scavenging enzymes such as glutathione peroxidase 1 (GPX1) and NRF2 signaling [46].

Inhibiting glutaminolysis enhances oxidative stress in combined treatment for cancer [49]. In contrast, in the Th17-skewing test, rosiglitazone and pioglitazone inhibit glutaminolysis but not glycolysis, decreasing GSH level and increasing ROS generation [49]. Additionally, oxidative stress may enhance glutaminolysis [41] to synthesize GSH to reduce oxidative stress. Therefore, glutaminolysis and oxidative stress provide a reciprocal regulation for each other (Figure 1).

### 2.5. Relationship between Fatty Acid Metabolism and Oxidative Stress

Modulating fatty acid metabolism can regulate oxidative stress. Mitochondrial fatty acid oxidation, a non-electron transfer chain (ETC) reaction, is accompanied by inducing oxidative stress [50,51]. Overexpressing acetyl-CoA carboxylase 1 (ACC1), a priming enzyme for fatty acid synthesis, shows antiproliferation and oxidative stress induction in a primary bone marrow culture [52]. In contrast, inhibiting ACC1 causes NADPH accumulation and decreases oxidative stress.

Different fatty acid metabolic enzymes have different responses or effects on oxidative stress. Inhibition of fatty acid synthase (FASN) stimulates oxidative stress to cause antiproliferation of breast cancer cells [51]. Inhibiting fatty acid transport protein 2 (FATP2) induces lipid production, decreases oxidative stress, and inhibits cancer stem cell proliferation [53]. Therefore, fatty acid metabolism and oxidative stress are related to reciprocal regulation (Figure 1).

### 2.6. Relationship between OXPHOS and Oxidative Stress

During OXPHOS, electron transfer occurs in the ETC, but it is usually accompanied by the leakage of mitochondrial superoxide, which is the main pool for oxidative stress in many cell types [54]. Inhibition of OXPHOS induces more oxidative stress attributed to electron accumulation in ETC, causing electron leakage, ROS production [55,56,57,58], and ATP depletion [59]. Examples of ETC inhibitors (rotenone, antimycin A, and carbonyl cyanide-p-trifluoromethoxyphenylhydrazone (FCCP)) show ROS and mitochondrial superoxide generation [60]. Therefore, OXPHOS and oxidative stress offer reciprocal regulation between each other (Figure 1).

## 3. PI3K/AKT/mTOR and Its Associated Metabolisms

**Figure 2:**
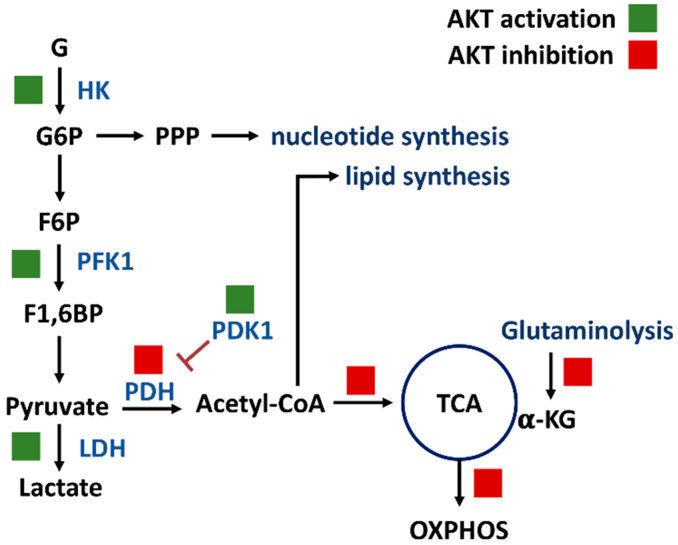
PI3K/AKT/mTOR signaling regulates metabolisms of glycolysis, PPP, nucleotide synthesis, lipid synthesis, TCA cycle, glutaminolysis, and OXPHOS. Solid and blank boxes indicate activation and inactivation by AKT. Abbreviations: G, glucose; G6P, glucose-6-phosphate; F6P, fructose-6-phosphate; F1,6BP, fructose-1,6-bisphosphate; HK, hexokinase; PFK1, phosphofructokinase 1; PDH, pyruvate dehydrogenase; PDK1, pyruvate dehydrogenase kinase 1; α-KG, α-ketoglutarate; TCA, tricarboxylic acid cycle; OXPHOS, oxidative phosphorylation.

**Figure 3:**
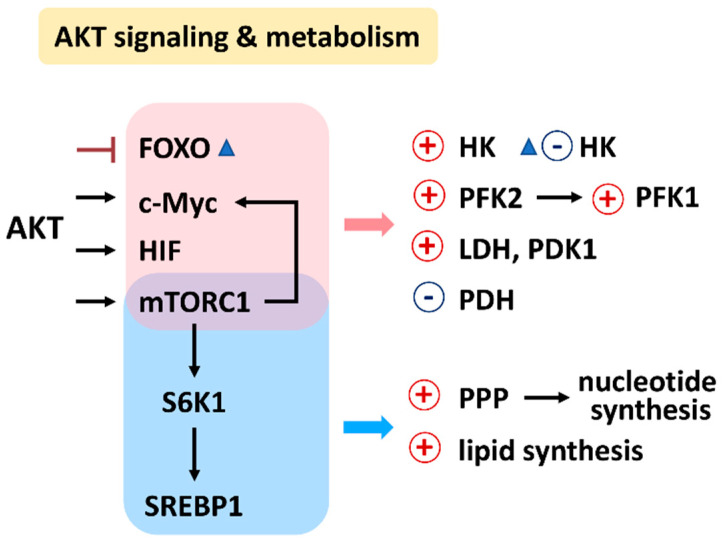
Relationship between AKT signaling, target enzymes, and their affected metabolisms. Abbreviations: FOXO, forkhead box transcription factors; HIF, hypoxia-inducible factor; mTORC1, mechanistic target of rapamycin complex 1; S6K1, mTOR substrate S6 kinase 1; sterol regulatory element-binding transcription factor 1; SREBP1; HK, hexokinase; PFK1/2, phosphofructokinase 1/2; PDK1, pyruvate dehydrogenase kinase 1; LDH, lactate dehydrogenase; PDH, pyruvate dehydrogenase; PPP, pentose phosphate pathway.

PI3K/AKT/mTOR signaling regulates a group of metabolisms such as glycolysis, PPP, nucleotide synthesis, lipid synthesis, TCA cycle, glutaminolysis, and OXPHOS (Figure 2) [7]. Moreover, the downstream effectors of the PI3K/AKT/mTOR, such as forkhead box transcription factors (FOXO), c-Myc, hypoxia-inducible factor (HIF), mechanistic target of rapamycin complex 1 (mTORC1), mTOR substrate S6 kinase 1 (S6K1), and sterol regulatory element-binding protein 1 (SREBP1), were reported. The target metabolic enzymes of PI3K/AKT/mTOR effectors were provided (Figure 3). In general, c-Myc, HIF, and mTORC1 activate hexokinase (HK), phosphofructokinase-2 (PFK2), PFK1, and lactate dehydrogenase (LDH) but inactivate pyruvate dehydrogenase (PDH) [7]. In contrast, AKT suppresses FOXO to inactivate HK, glucose-6-phosphate isomerase (GPI), aldolase, enolase, and pyruvate kinase (PK), leading to activate glycolysis and generating pyruvate. Moreover, mTORC1, S6K1, and SREBP1 activate the PPP pathway and lipid synthesis [7]. Therefore, PI3K/AKT/mTOR signaling is crucial for regulating different metabolisms.

**Figure 4:**
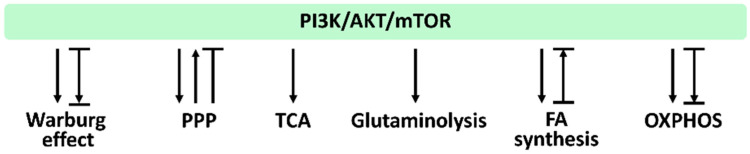
PI3K/AKT/mTOR and its associated metabolisms. Arrows indicate activation; T indicates inhibition; T with an arrow indicates PI3K/AKT/mTOR inhibition leading to metabolic activation. Abbreviations: PPP, pentose phosphate pathway; TCA, tricarboxylic acid cycle; FA, fatty acid; OXPHOS, oxidative phosphorylation. These effects were summarized from the reports mentioned in Section 3.1, Section 3.2, Section 3.3, Section 3.4, Section 3.5, Section 3.6. Different studies reported differential regulations to these metabolisms by modulating PI3K/AKT/mTOR signaling. Various reports show different PI3K/AKT/mTOR responses for the same metabolism.

In Section 3.1, Section 3.2, Section 3.3, Section 3.4, Section 3.5, Section 3.6, we provide the impact of PI3K/AKT/mTOR signaling on regulating their associated metabolisms, such as the Warburg effect, PPP, TCA cycle, glytaminogenesis, fatty acid synthesis, and OXPHOS, which is summarized in Figure 4.

### 3.1. Relationship between the Warburg Effect and PI3K/AKT/mTOR

The PI3K/AKT/mTOR axis is a tightly connected pathway starting at PI3K and subsequently activates AKT and mTOR. Some studies report parts of such pathways but do not exclude participation in others.

Several studies examined the function of PI3K/AKT/mTOR in the Warburg effect by upregulation or downregulation strategies. Overexpressing the activated AKT increases glucose uptake by activating the glucose transporter GLUT1 [61] and enhances the Warburg effect [61]. In contrast, inhibiting AKT/mTOR/GLUT1 signaling by berberine can suppress the Warburg effect for antiproliferation of breast and liver cancer cells [62]. Accordingly, modulating the PI3K/AKT/mTOR axis regulates the Warburg effect (Figure 4).

### 3.2. Relationship between PPP and PI3K/AKT/mTOR

Several studies examined the function of PI3K/AKT/mTOR in PPP by modulating their protein or enzyme levels and activities. Glucose-6-phosphate dehydrogenase (G6PD), a priming enzyme of PPP, is stabilized by PI3K/*AKT* activation to promote PPP [63]. Suppressing the expression of the pleckstrin homology like domain family A member 3 (PHLDA3), an intrinsic AKT inhibitor, can improve the PI3K activation and switch glycolysis to PPP [63].

Transketolase (TKT), one of the PPP enzymes, is highly expressed in colorectal cancer, giving a poor prognosis [64]. TKT is also upregulated in colorectal cancer cell lines, promoting proliferation and metastasis. TKT overexpression induces AKT activation [64]. Accordingly, PPP may activate AKT. However, PPP may have a different response to AKT. PPP can inactivate AKT to induce antiproliferation of neuroblastoma cells [65]. Therefore, PPP and PI3K/AKT/mTOR offer reciprocal regulation (Figure 4).

### 3.3. Relationship between the TCA Cycle and PI3K/AKT/mTOR

The PDH complex catalyzes the reaction converting pyruvate to acetyl-CoA. Then, acetyl-CoA joints oxoacetate to enter the TCA cycle and becomes citrate. PI3K activation can regulate the Warburg effect, partly inhibit pyruvate kinase 2 (PKM2), a rate-limited enzyme of glycolysis, and finally dissociates the connection between glycolysis and the TCA cycle [63]. PDH activity is suppressed by pyruvate dehydrogenase kinase (PDK1) [18]. AKT can activate PDK1 by phosphorylation to enhance its PDH inhibition for pausing the TCA cycle and switching to the LDH response in the Warburg effect [18]. Accordingly, PI3K/AKT/mTOR shows a close relationship to TCA cycle regulation (Figure 4).

Moreover, the metabolites at the pausing TCA cycle also change to other pathways such as lipid synthesis [66]. mTOR involved in glutaminolysis contributing to TCA cycle regulation is described later [67]. Therefore, PI3K/AKT/mTOR signaling functions as a TCA modulator.

### 3.4. Relationship between Glutaminolysis and PI3K/AKT/mTOR

Glutaminolysis is connected to the TCA cycle at the entry of α-ketoglutarate, fueling the TCA cycle [66]. Mitochondrial pyruvate carrier (MPC) can transport pyruvate from the cytoplasm to mitochondria. Inhibiting MPC activates glutamate dehydrogenase (GDH), which converts glutamate to α-ketoglutarate, and generates acetyl-CoA from glutamine [66]. Accordingly, MPC inhibition changes the paths to replenish TCA intermediates [66]. Since PI3K/AKT/mTOR regulates the TCA cycle, it also controls glutaminolysis.

Cancer cells highly express PI3K-*AKT*-mTOR and improve glutaminolysis [68]. NAD(P)H: quinone oxidoreductase 1 (NQO1) is an antioxidant signaling protein. In NQO1-defective liver cancer cells, both glycolysis and glutaminolysis-associated gene expressions are suppressed by AKT [69]. In contrast, NQO1 overexpression induces PI3K/AKT activation to improve liver cancer cell proliferation. Therefore, PI3K/AKT/mTOR function as modulators for controlling glutaminolysis (Figure 4).

### 3.5. Relationship between Fatty Acid Metabolism and PI3K/AKT/mTOR

Several studies examined the function of PI3K/AKT/mTOR in the gene expressions for fatty acid metabolism. ATP citrate lyase (ACLY) catalyzes the conversion of TCA cycle-derived citrate to acetyl-CoA in the cytoplasm. AKT can activate ACLY by phosphorylation [70] to control fatty acid synthesis [18], providing de novo lipid synthesis. PI3K/AKT/mTOR is the upstream regulator for the melanoma antigen ganglioside GD3. GD3 can activate SREBP1 and, in turn, regulates ACC1 expression [71].

Upregulation of human epidermal growth factor receptor 2 (HER2) in breast cancer cells enhances the expression of fatty acid synthesis genes such as ACC1 and FASN, which are suppressed by PI3K and mTOR inhibitors, indicating that PI3K/AKT/mTOR can regulate fatty acid synthesis [72]. AKT/mTOR is overexpressed in liver cancer cells and induces upregulation of lipogenesis [73]. In ACC2 knockdown mice, fatty acid synthesis-associated genes, including ACC1, FASN, and ATP citrate lyase (ACL), are downregulated [74]. Therefore, PI3K/Akt/mTOR plays a vital role in regulating fatty acid metabolism (Figure 4).

### 3.6. Relationship between OXPHOS and PI3K/AKT/mTOR

AKT activation enhances OXPHOS in both normal and cancer cells. After a nephrotoxic injury in renal proximal tubular cells, AKT is activated [75]. ETC activity and ATP generation rate are the critical indicators for OXPHOS, which is proportional to oxygen consumption rate (OCR). PI3K/AKT/mTOR pathway inhibition suppresses OCR in head and neck cancer cells [76]. Therefore, PI3K/AKT/mTOR plays a vital role in regulating OXPHOS metabolism (Figure 4).

## 4. Interaction between Oxidative Stress and PI3K/AKT/mTOR

**Figure 5:**
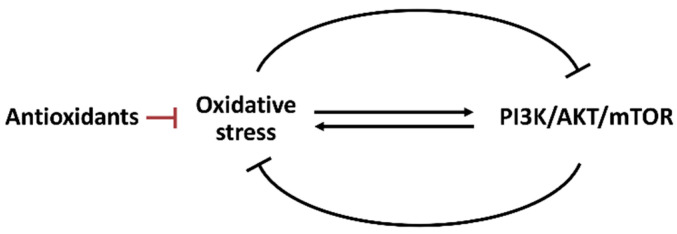
Interaction between oxidative stress and PI3K/AKT/mTOR. Oxidative stress and PI3K/AKT/mTOR can reciprocally induce or suppress each other. The antioxidant system also regulates oxidative stress.

Oxidative stress and oncogene signal transduction are essential in regulating metabolism [77]. Oxidative stress and PI3K/AKT/mTOR pathways show the reciprocal modulation of several cell stress responses such as apoptosis [78], autophagy [79], senescence [79,80], and ER stress [81]. Oxidative stress may regulate the PI3K/AKT/mTOR activity (Figure 5). Hydrogen peroxide inactivates protein tyrosine phosphatase 1B (PTP1B) to inhibit PI3K. Hydrogen peroxide also inactivates protein phosphatase 2A (PP2A) to inhibit AKT [18].

Under moderate levels of ROS, AKT activation happens [79] through PI3K signaling [82]. However, ROS may dephosphorylate and inactivate PI3K/AKT of gastric cancer cells following the treatment of the thioredoxin reductase-1 inhibitor chaetocin [78].

In contrast, PI3K/AKT/mTOR may regulate oxidative stress (Figure 5). Phosphatase and tensin homologous (PTEN) proteins suppress PI3K signaling [79]. Hence, in PTEN-deficient prostate cancer cells, AKT is hyperactivated to highly induce ROS generation attributed to OXPHOS induction [83]. Growth factor activates AKT to enhance ROS generation, leading to uncontrolled cancer cell proliferation [84]. Notably, PI3K/AKT/mTOR may regulate oxidative stress differently. PI3K/AKT is activated in dental pulp cells at hypoxia to suppress oxidative stress [85].

Moreover, redox homeostasis is balanced between oxidative stress and the antioxidant system. AKT activation improves oxidative stress adaptation by activating NRF2-associated antioxidant signaling [18]. Hence, oxidative stress and PI3K/AKT/mTOR show the complex interaction in redox homeostasis. Due to reciprocal regulation, drug treatments that directly affect one may indirectly influence the other. Accordingly, oxidative stress and PI3K/AKT/mTOR exhibit multiple functions regulating their respective metabolisms.

## 5. The Roles of Oxidative Stress and PI3K/AKT/mTOR in Fucoidan

Several bioactive compounds have been identified in algae [86,87,88,89,90]. Fucoidan is a brown algae-derived polysaccharide capable of generating abundant sulfated fucoses [91]. Fucoidan has been isolated from several brown algae, including *Alaria esculenta*, *Ascophyllum nodosum*, *Cladosiphon okamuranus*, *Colpomenia sinuosa*, *Fucus vesiculosus*, *Fucus evanescens*, *Ecklonia cava*, *Hizikia fusiforme*, *Laminaria hyperborea*, *Laminaria japonicia*, *Macrocystis pyrifera*, *Saccharina japonica*, *Sargassum confusum*, *Sargassum coreanum*, *Sargassum filipendula*, *Sargassum horneri*, *Sargassum mcclurei*, *Sargassum natans*, *Sargassum polycystum*, and *Sargassum siliquastrum* [92,93,94,95,96,97,98,99,100,101,102,103].

Fucoidan is a safe food supplement authorized by the United States Food and Drug Administration (FDA) [25]. Several bioactivities of fucoidan have been reported in suppressing inflammation, coagulant, microbial infection, and oxidation [104]. Moreover, some studies focus on the chemopreventive effects of fucoidan [98,105,106,107,108,109]. Since most of these bioactivities, such as inflammation [110], chemoprevention [111], and anticancer [112] effects, primarily rely on the modulation of oxidative stress, we discuss the development of oxidative stress for fucoidan research in Section 5.1.

Moreover, the anticancer effects of fucoidan were emphasized in several reviews [92,113,114,115]. Mounting evidence shows that fucoidan exhibits antiproliferation effects against several types of cancers, such as cholangiocarcinoma [116], breast [117,118], pancreas [119], lung [120,121], and cervical [122] cancer cells. Since these anticancer effects are associated with modulating PI3K/AKT/mTOR [123,124,125,126,127,128], PI3K/AKT/mTOR signaling of fucoidan-related studies is also summarized in Section 5.2.

### 5.1. Oxidative Stress Studies of Fucoidan

Exogenous antioxidants are capable of biphasic functions for modulating oxidative stress, i.e., decreasing and increasing oxidative stress at normal and lethal concentrations [129]. Fucoidan from *Undaria pinnatifida* [130] and *Sargassum filipendula* [131] show biochemical antioxidant effects by examining 2,2-diphenyl-1-picrylhydrazyl (DPPH) scavenging ability. Several studies reported that fucoidan exhibits chemopreventive effects on hazardous chemicals, radiation, and toxins, while others reported antiproliferative effects against cancer cells.

Given the chemopreventive effects, fucoidan exhibits protective effects against acetaminophen-triggered hepatotoxicity by improving the expression and translocation of antioxidant NRF2, upregulating GSH, superoxide dismutase (SOD), and catalase (CAT), and suppressing ROS and lipid peroxidation (oxidative stress) of hepatocytes [105]. Similarly, fucoidan increases viability and decreases apoptosis, DNA damage, and ROS generation in UVB-irradiated human keratinocytes, accompanied by NRF2 activation [107]. Fucoidan also inhibits tumor necrosis factor (TNF)-α/interferon (IFN)-γ triggered inflammation and ROS generation of keratinocytes by turning on NRF2 signaling [98].

Moreover, fucoidan shows in vivo chemopreventative effects. Fucoidan increases survival and decreases oxidative stress and heart-beating induced by hydrogen peroxide in zebrafish embryos [132]. Besides NRF2 activation, the GSH level increases in fucoidan and high-fat diet-fed mice, accompanied by decreasing protein and lipid peroxidation [106].

Given the antiproliferation effects against cancer cells, fucoidan shows antiproliferation, oxidative stress, and apoptosis-inducing results in several kinds of cancer cells, such as breast [133], liver [134], lung [135], and colon [136]. Since redox homeostasis is the outcome of balancing oxidative stress and the antioxidant system, it is possible that the antioxidant system is down-regulated and subsequently induces oxidative stress. Therefore, fucoidan can modulate oxidative stress to protect normal cells or kill cancer cells (Table 1).

### 5.2. PI3K/AKT/mTOR Studies of Fucoidan

Fucoidan decreases phosphor-PI3K/AKT to inactivate PI3K/AKT and induce apoptosis in acute promyelocytic leukemia [123], lung [124], prostate [125], liver [126], bladder [127], and colon [128] cancer cells. Fucoidan also shows preferential killing of ovarian cancer cells but not normal ovarian epithelial cells by downregulating cancer cell PI3K/AKT signaling [137]. In a 7,12-dimethylbenz[a]anthracene (DMBA)-induced animal tumor model, fucoidan suppresses breast cancer cell-xenografted tumor growth by inhibiting PI3K/AKT/GSK3β signaling [138]. Moreover, fucoidan shows anti-migration and anti-invasion in lung [139] and colon [128] cancer cells by downregulating PI3K/AKT/mTOR signaling. Therefore, fucoidan induces several PI3K/AKT/mTOR-mediated responses (Table 1).

## 6. The Roles of Fucoidan-Modulated Oxidative Stress and PI3K/AKT/mTOR in Metabolic Regulations

The relationship between fucoidan-modulated oxidative stress and PI3K/AKT/mTOR in metabolic regulations is summarized in Table 1. As mentioned in Section 2 and Section 3, oxidative stress and PI3K/AKT/mTOR show the modulating effects on these mechanisms. Fucoidan shows the regulation of oxidative stress and PI3K/AKT/mTOR. However, the impact of fucoidan-modulated oxidative stress and PI3K/AKT/mTOR on these mechanisms remain unclear. We next discuss the potential role of oxidative stress and PI3K/AKT/mTOR in the metabolic regulation of fucoidan.

### 6.1. The Roles of Fucoidan-Induced Oxidative Stress in Metabolic Regulations Need Further Investigation

Fucoidan shows antiproliferation, apoptosis, and oxidative stress-related responses on several cancer cells. However, the impact of fucoidan-induced oxidative stress on regulating metabolism was not thoroughly investigated (Table 1). Fucoidan shows antiproliferation effects on liver cancer cells by triggering oxidative stress generation and apoptosis, accompanied by GSH depletion [134]. Fucoidan also sensitizes breast cancer cells to anticancer drugs such as cisplatin, tamoxifen, or paclitaxel by downregulating GSH levels [140]. Therefore, fucoidan provides oxidative stress-dependent antiproliferation to cancer cells (Table 1). However, the role of oxidative stress in regulating the metabolism of fucoidan treatment lacks detailed investigation.

### 6.2. The Roles of Fucoidan-Inactivated PI3K/AKT/mTOR in Metabolic Regulations Need Further Investigation

Fucoidan also shows PI3K/AKT/mTOR inactivation-related responses on several cancer cells. However, the impact of PI3K/AKT/mTOR signaling on regulating the metabolism of fucoidan treatment was not thoroughly investigated (Table 1).

Several fatty acid metabolism studies affected by fucoidan were reported (Table 1); however, the roles of oxidative stress and PI3K/AKT/mTOR in regulating fatty acid metabolism remain unclear. Fucoidan inhibits proliferation by inhibiting fatty acid synthesis (ACC) in hepatoma HLF cells [141]. Moreover, fucoidan suppresses HMG-CoA reductase (HMGCR) and improves lecithin-cholesterol acyltransferase (LCAT) expressions, and reduces cholesterol synthesis [142]. Fucoidan also suppresses SREBP1c expression and reduces fatty acid synthesis. It enhances the peroxisome proliferator-activated receptor α (PPARα), PPARγ, and lipoprotein lipase (LPL) expression to drive the β-oxidation reaction of fatty acids [142]. Similarly, fucoidan activates lipolysis enzymes, e.g., hormone-sensitive lipase (HSL), to decrease lipid storage in adipocytes [143].

Except for the fatty acid metabolism, the remaining metabolisms such as Warburg Effect, PPP, TCA cycle, glutaminolysis, and OXPHOS were not connected to fucoidan studies. Moreover, fucoidan can modulate oxidative stress and PI3K/AKT/mTOR signaling. Nevertheless, the contributions of fucoidans to regulating the Warburg Effect, PPP, TCA cycle, glutaminolysis, fatty acid metabolism, and OXPHOS remain unstudied. This holds particularly for a detailed examination of the effects that oxidative stress and PI3K/AKT/mTOR may provide in regulating the Warburg Effect, PPP, TCA cycle, glutaminolysis, fatty acid metabolism, and OXPHOS of fucoidan (Table 1).

## 7. Conclusions

Metabolism is at the center of cancer cell proliferation. Oxidative stress and PI3K/AKT/mTOR signaling play crucial roles in controlling metabolism for carcinogenesis. The relationships between oxidative stress and PI3K/AKT/mTOR signaling and individual metabolism were summarized. These signaling pathways exhibit a diverse regulation of different metabolisms of cancer cells. As mentioned above, oxidative stress and PI3K/AKT/mTOR signaling are well organized and connected to several metabolisms in cancer cells. They may reroute when some of them are suppressed. The relationships between the Warburg effect, PPP, fatty acid metabolism, TCA cycle, glutaminolysis, and OXPHOS were demonstrated here.

Accordingly, natural products or other chemical agents exhibiting oxidative stress and PI3K/AKT/mTOR modulating functions may potentially regulate cancer cell development. This review chose the brown algae-derived fucoidan to discuss its impact on oxidative stress and PI3K/AKT/mTOR and their modulating effects on metabolisms. Although fucoidan impacts oxidative stress and PI3K/AKT/mTOR signaling, their possible regulating metabolisms remain unclear.

**Figure 6:**
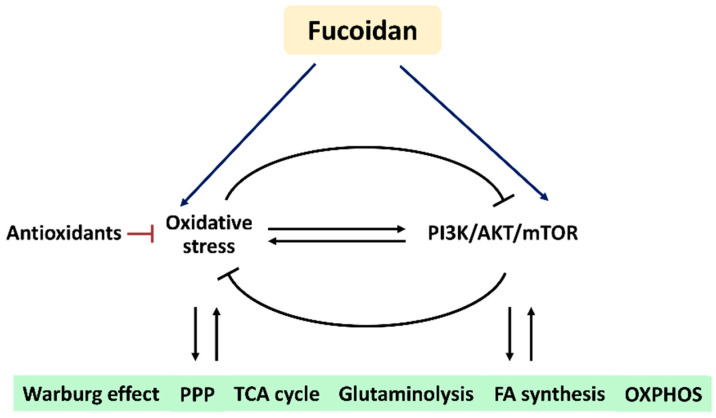
Hypothesis. Fucoidan may modulate oxidative stress and PI3K/AKT/mTOR in metabolic regulations. Oxidative stress and PI3K/AKT/mTOR can reciprocally induce or suppress each other. The antioxidant system also regulates oxidative stress. Fucoidan can modulate oxidative stress and PI3K/AKT/mTOR, but their impacts on fucoidan-modulated metabolisms are rarely investigated. Accordingly, fucoidan may trigger oxidative stress and PI3K/AKT/mTOR to control several metabolic functions. Abbreviations: PPP, pentose phosphate pathway; TCA, tricarboxylic acid cycle; FA, fatty acid; OXPHOS, oxidative phosphorylation.

Based on these findings, we hypothesize that fucoidan regulates oxidative stress and PI3K/AKT/mTOR signaling to modulate their associated metabolic regulations (Figure 6). Understanding this connection and mechanism may provide a novel strategy to investigate the roles of oxidative stress and PI3K/AKT/mTOR signaling in metabolism-based cancer therapy using fucoidan in the future.

Notably, these metabolisms crosstalk with each other, and they receive integrating effects from oxidative stress and PI3K/AKT/mTOR signaling. Using the inhibitors or activators of these metabolisms may provide a deep understanding of the metabolism functions of drug-modulating changes on oxidative stress and PI3K/AKT/mTOR signaling. Moreover, the combined treatments with some of these metabolic modulators may also improve the anticancer therapeutic effects. In addition to fucoidan, other anticancer agents with the modulating ability of oxidative stress and PI3K/AKT/mTOR signaling may use the same strategy to enhance their antiproliferation effects on cancers.

Therefore, the contribution of this review is to shed light on the existing knowledge of individual and interacting effects of oxidative stress and PI3K-AKT-mTOR and provide an effective strategy for applying these metabolism-related regulations in cancer therapy.

## Figures and Tables

**Table 1:** Relationship between fucoidan-modulated oxidative stress and PI3K/AKT/mTOR in metabolic regulations *.

	Warburg Effect	PPP	TCA Cycle	Glutaminolysis	Fatty Acid Synthesis	OXPHOS
Oxidative stress	●	●	●	●	●	●
PI3K/AKT/mTOR	●	●	●	●	●	●
Fucoidan	○	○	○	○	●	○
Fucoidan Oxidative stress	○	○	○	○	○	○
Fucoidan PI3K/AKT/mTOR	○	○	○	○	○	○

* When literature support is available, this is marked with “●”, otherwise it shows “○”.
